# AI empowerment and supervisory board effectiveness: Evidence from an information processing framework

**DOI:** 10.1371/journal.pone.0360152

**Published:** 2026-10-08

**Authors:** Ruisheng Qin, Jiaxing Qin, Shiquan Wang

**Affiliations:** 1 School of Business Administration, Northeastern University, Shenyang, China; 2 School of Accounting, Guangxi University of Finance and Economics, Nanning, China; 3 Personnel Office, Guangxi International Business Vocational College, Nanning, China; Otago Polytechnic, NEW ZEALAND

## Abstract

In the context of rapid digitalization and intelligent governance, artificial intelligence (AI) technologies are increasingly embedded in organizational supervision and decision-making processes, fundamentally reshaping how supervisory boards process information and perform monitoring tasks. Drawing on information processing theory, this study develops a moderated mediation model to examine how AI empowerment is associated with supervisory board effectiveness through information transparency, and how this process is contingent upon organizational learning capability. Using survey data collected from 382 supervisory board members and senior managers across multiple industries, this study adopts an organizational-level perspective and employs hierarchical regression and bootstrap analyses to test the proposed hypotheses. The results show that AI empowerment significantly associated with both information transparency and supervisory board effectiveness. Information transparency partially mediates the relationship between AI empowerment and supervisory effectiveness, indicating that AI is positively associated with supervisory performance by optimizing organizational information-processing mechanisms. Moreover, organizational learning capability positively moderates the relationship between AI empowerment and information transparency. However, the moderated mediation effect is not statistically significant, indicating that the indirect effect of AI empowerment on supervisory effectiveness through information transparency remains relatively stable across different levels of organizational learning capability. These findings suggest that organizational learning capability shapes the relationship between AI empowerment and information transparency, but does not significantly alter the overall indirect pathway to supervisory effectiveness.

## 1. Introduction

In the era of the digital economy and intelligent governance, the widespread application of Artificial Intelligence (AI) is profoundly reshaping the operational logic and supervisory mechanisms of corporate governance [1]. As a key supervisory body within the corporate governance system, the supervisory board plays an essential role in mitigating agency problems, safeguarding shareholders’ interests, and ensuring sustainable corporate development. However, traditional supervisory models often rely on human judgment, experiential analysis, and qualitative assessment. These approaches are constrained by limited information sources, slow processing speed, and insufficient analytical depth, making it difficult to meet the governance demands of firms operating in highly complex and uncertain environments [2]. With the exponential growth of corporate data, the information-processing burden associated with supervision has sharply increased. If supervisory boards fail to effectively integrate and analyze complex information, they may face supervision delays, judgmental biases, and governance failures [3]. Against this backdrop, AI empowerment has emerged as a crucial technological pathway for enhancing supervisory board effectiveness. By leveraging machine learning, natural language processing, and intelligent analytics, AI enables the rapid collection, automatic identification, and multidimensional analysis of information, thereby providing supervisory boards with more comprehensive, accurate, and forward-looking decision support [4]. AI not only reduces human subjectivity but also significantly associated with the scientific rigor and efficiency of supervisory decisions, injecting new momentum into the construction of high-quality corporate governance systems [5].

Nevertheless, AI empowerment does not automatically lead to performance improvement. Existing studies reveal considerable variation in governance performance outcomes following AI adoption. Some firms have successfully achieved digital and intelligent transformations of their supervisory processes, while others have fallen into a “technology in form only” dilemma, where AI applications remain superficial and fail to substantially enhance supervisory effectiveness [6,7]. This discrepancy suggests that the effects of AI empowerment are not unidirectional or linear; rather, specific mediating mechanisms and boundary conditions may shape its impact on supervisory performance. In other words, the adoption of AI serves merely as a means. Whether it truly associated with supervisory effectiveness depends on whether the organization’s internal information processing mechanisms are optimized and whether it possesses sufficient capability to learn and assimilate new technologies [8]. Consequently, understanding how AI empowerment affects supervisory performance, through what mechanisms it operates, and under what conditions its effects are most salient has become an important issue requiring systematic investigation.

To explore these relationships, this study adopts Information Processing Theory (IPT) as the theoretical foundation [9]. The central premise of IPT is that organizational performance depends on the alignment between task complexity and information processing capacity [10]. When task complexity exceeds an organization’s information processing capability, information asymmetry and decision biases arise, ultimately leading to efficiency losses or supervisory failures. Conversely, when organizations possess sufficient information processing capacity, they can better identify, transmit, and integrate critical information, thereby improving decision-making and supervisory performance [9]. Within this theoretical framework, supervisory activities can be viewed as tasks characterized by high information-processing demands, requiring the supervisory body to identify potential risks, evaluate managerial behaviors, and propose corrective recommendations from large volumes of unstructured information [11]. AI empowerment plays a pivotal role in enhancing information processing capability in this context. Through automated data collection, intelligent filtering, and algorithmic analysis, AI significantly increases the speed and depth of information identification and processing, thereby strengthening the informational foundation for decision-making and improving supervisory efficiency.

From the perspective of information processing theory, information transparency plays a central mediating role in this process. Information transparency reflects the openness and accuracy of information generation, transmission, and sharing within organizations, serving as an indicator of the efficiency and quality of organizational information systems [12]. The adoption of AI enables more automated and traceable data collection and disclosure, allowing supervisory boards to access real-time operational and risk-related information. This reduces information asymmetry and positively associated with the relevance and timeliness of supervisory activities [13]. Higher levels of information transparency provide supervisory boards with a more solid informational foundation for monitoring managerial behavior, enabling them to detect anomalies and risks more accurately and to propose data-driven supervisory recommendations [14,15]. Therefore, it can be inferred that AI empowerment associated with supervisory performance indirectly by enhancing information transparency and optimizing the information processing mechanism. In other words, information transparency serves as a key mediating channel through which AI empowerment translates into improved supervisory performance.

Furthermore, technological empowerment does not automatically translate into improved organizational performance; rather, the key lies in the organization’s ability to transform technological potential into practical governance capability through learning. Within this logic, organizational learning capability emerges as an important moderating factor influencing the relationship between AI empowerment and supervisory performance [16–18]. Organizational learning capability refers to the systematic ability of an organization to acquire, absorb, integrate, and apply new knowledge. It reflects the willingness and adaptability of organizational members to learn and adjust to new technologies, processes, and knowledge [19,20]. Organizations with strong learning capability can more rapidly understand the logic of AI tools and proactively explore their applications in supervisory contexts, thereby converting improvements in information processing into tangible supervisory effectiveness [21]. Conversely, organizations with weak learning capability, even when equipped with advanced AI systems, may suffer from a “technology–institution mismatch” due to the lack of mechanisms for knowledge absorption and reinvention, which substantially weakens the potential of AI empowerment [22,23]. Therefore, organizational learning capability not only moderates the relationship between AI empowerment and information transparency but also amplifies the mediating pathway through which AI is positively associated with supervisory performance via information transparency.

It is important to clarify the level of analysis adopted in this study. Theoretically, this research is anchored at the organizational level, focusing on how AI empowerment shapes supervisory board effectiveness through organizational information-processing mechanisms. Although data are collected from individual supervisory board members and senior managers, these respondents serve as key informants providing assessments of collective governance practices rather than reporting on their personal attitudes or behaviors. Furthermore, aggregation analyses reported later in this study confirm sufficient within-group agreement and between-group reliability, supporting the treatment of these assessments as organizational-level constructs. Accordingly, all core constructs in this study—AI empowerment, information transparency, organizational learning capability, and supervisory board effectiveness—are conceptualized as organizational-level phenomena. Building on this reasoning, the present study adopts information processing theory as the overarching analytical framework and develops a moderated mediation model to examine how AI empowerment influences supervisory board performance through information transparency and how organizational learning capability moderates this process. This model contributes to corporate governance research by deepening the information-processing perspective and offering a new explanatory pathway for understanding the mechanisms through which AI technologies function in organizational supervision.

This study makes several important contributions to the literature on corporate governance and intelligent governance. First, this study extends prior research by theorizing and empirically examining AI empowerment as an organizational-level information-processing capability that is positively associated with supervisory board effectiveness, thereby broadening the application of information processing theory to the context of AI-enabled corporate supervision. Second, this study identifies information transparency as a critical mediating mechanism through which AI empowerment is positively associated with supervisory board effectiveness, revealing the internal information-processing pathway linking AI technologies to governance performance. Third, by introducing organizational learning capability as a moderating variable, this study examines the boundary condition under which AI empowerment is associated with information transparency and further tests whether this moderation extends to the indirect effect on supervisory effectiveness. Collectively, these contributions deepen the understanding of how AI empowerment operates within corporate governance systems and offer novel theoretical and empirical insights into the interplay between technological empowerment, organizational capabilities, and supervisory performance.

## 2. Theoretical foundation and hypothesis development

### 2.1 Theoretical foundation

Information Processing Theory (IPT) posits that organizational performance depends on the degree of alignment between task complexity and information processing capacity. In this study, IPT is applied at the organizational level, where information processing capacity is viewed as a collective capability embedded in governance structures and supervisory systems, rather than as an individual-level cognitive process. When organizations face complex and uncertain external environments, insufficient information processing capability can lead to information asymmetry, cognitive bias, and decision errors. Conversely, enhanced information processing capability can effectively reduce uncertainty, improve decision quality, and strengthen supervisory performance [9]. Information processing capability is reflected in the efficiency and accuracy of an organization’s information collection, transmission, analysis, and utilization processes. The emergence of AI technologies has significantly expanded organizational information-processing boundaries, enabling faster and more precise information recognition and analysis, thereby providing the supervisory board with new technological support and decision-making foundations. At the same time, information processing theory emphasizes that the effectiveness of technological tools depends on an organization’s ability to learn and absorb new information. Only when organizations possess strong learning capability and can actively assimilate and transform the knowledge and insights provided by AI can the full potential of information processing be realized [9].

### 2.2 AI empowerment and information transparency

When organizations face highly complex and uncertain governance environments, insufficient information processing capability can result in distorted information and biased decisions, thereby weakening supervisory effectiveness. AI empowerment refers to the application of AI technologies in governance and supervision processes, using algorithmic analysis, machine learning, and natural language processing to enhance organizations’ abilities to recognize, collect, analyze, and integrate information [24]. AI empowerment is not merely a technological investment but an expansion of information-processing capability. It enables supervisory boards to efficiently obtain and analyze complex information when performing their oversight tasks [25]. AI systems can automatically detect operational risks, integrate multisource data, and generate interpretable insights, allowing supervisory board members to obtain comprehensive and accurate governance information in a shorter time [26]. This enhanced capability is positively associated with the organization’s internal information-processing mechanism and strengthens the quality of information flows.

In this process, information transparency serves as a key manifestation of AI empowerment. Information transparency refers to the openness, accuracy, and symmetry of information generation, transmission, and sharing within organizations. It reflects the efficiency and quality of organizational information systems [27]. High information transparency allows supervisory boards to more effectively access and interpret critical data and operational conditions, reducing information asymmetry and laying a solid foundation for effective supervision [28]. Through data integration and real-time analysis mechanisms, AI empowerment facilitates smoother information flows, more timely disclosures, and greater data traceability, thereby significantly enhancing information transparency. For instance, AI-based financial monitoring systems can automatically identify abnormal transactions, generate reports, and share them with supervisory bodies, thus reducing the delays and omissions caused by manual screening [29]. This process exemplifies how AI empowerment is positively associated with organizational information-processing capability, which is concretely manifested in improved information transparency [30]. In summary, the stronger the AI empowerment, the more efficient the organization’s information-processing mechanism, and the more open and symmetrical its internal information flows become. Based on this reasoning, the following hypothesis is proposed:

H1: AI empowerment has a significant positive effect on information transparency.

### 2.3 Information transparency and supervisory board effectiveness

Information transparency is a crucial indicator of an organization’s information-processing capability, reflecting the openness, accuracy, and symmetry in its information generation, transmission, and sharing processes [31]. High levels of information transparency facilitate smooth internal information flows, timely feedback, and adequate disclosure, allowing supervisory bodies to gain a comprehensive understanding of operational conditions and potential risks, thereby reducing information asymmetry [32]. For supervisory boards, information transparency constitutes the fundamental condition for effective monitoring. Only when supervisory boards can obtain timely and accurate information on corporate operations and finances can they detect potential misconduct, identify managerial opportunism, and propose targeted governance recommendations [33]. Conversely, when internal information systems are closed, disclosures incomplete, or communication ineffective, supervisory boards lack sufficient factual bases for decision-making, leading to superficial or delayed oversight [34].

Supervisory Board Effectiveness refers to the actual performance achieved by supervisory boards in fulfilling oversight responsibilities, mitigating agency risks, and improving governance decisions. It captures the board’s capacity and outcomes in information analysis, risk warning, problem detection, and recommendation implementation. High-performing supervisory boards typically rely on comprehensive information systems to conduct in-depth analyses, make evidence-based judgments, and promote the optimization of corporate governance structures [35]. Information processing theory suggests that when information transparency improves, internal informational uncertainty is significantly reduced, providing a more reliable basis for supervisory judgment, reducing decision biases and information distortion, and ultimately improving supervisory performance [9]. Particularly under AI empowerment, information disclosure becomes more timely and traceable, enabling supervisory boards to conduct forward-looking supervision and dynamic risk identification. Enhanced transparency strengthens the depth and accuracy of oversight and promotes the scientific and actionable quality of supervisory recommendations [14]. Therefore, based on this logic, the following hypothesis is proposed:

H2: Information transparency has a significant positive effect on supervisory board effectiveness.

### 2.4 The mediating role of information transparency

Supervisory activities represent a highly complex task that requires supervisory bodies to make judgments and decisions under conditions of multisource information and high uncertainty. When an organization’s information processing capability is inadequate, delays in information transmission, data distortion, and poor communication can undermine supervisory outcomes. As a key technological enabler that is positively associated with organizational information processing capability, AI empowerment strengthens the organization’s capacity for information recognition, collection, integration, and analysis through algorithmic learning, intelligent analytics, and data mining. AI applications not only expand the scope of information that organizations can process but also accelerate and refine the flow of information, making internal information-processing mechanisms more systematic and automated [25]. However, the effect of AI empowerment on supervisory performance is not direct but operates through specific information mechanisms that influence supervisory decision-making and behavior.

In this mechanism, information transparency plays a central mediating role. High information transparency enables supervisory boards to access critical governance information promptly and accurately, reducing information asymmetry, cognitive bias, and oversight blind spots [27]. AI empowerment is positively associated with information transparency by automating and digitizing the processes of data collection and disclosure, thereby reducing human interference and subjective bias [32]. In addition, AI-supported data analysis and visualization tools help supervisory boards interpret complex information more effectively, increasing the readability and decision-support value of data [31]. As transparency improves, supervisory boards can access more complete and reliable information, enhancing the precision and effectiveness of their oversight activities and improving the scientific quality and implementation of supervisory recommendations [34]. Supervisory Board Effectiveness, defined as the performance achieved by supervisory boards in fulfilling oversight duties, preventing agency problems, and promoting governance improvement, is thereby enhanced [12]. Higher information transparency enables supervisory bodies to conduct forward-looking, data-driven supervision, reducing passive post-event interventions and increasing both efficiency and depth [32].

In summary, AI empowerment is positively associated with supervisory performance indirectly by improving information transparency and optimizing the organization’s information-processing mechanism. Thus, information transparency mediates the relationship between AI empowerment and supervisory performance. Based on this reasoning, the following hypothesis is proposed:

H3: Information transparency mediates the relationship between AI empowerment and supervisory board effectiveness.

### 2.5 The moderating role of organizational learning capability

AI empowerment is positively associated with organizational information collection, integration, and analysis through algorithmic learning, intelligent identification, and data analytics, thereby improving information transparency. However, the effectiveness of AI technologies does not occur automatically. Their potential in information processing depends on the organization’s ability to understand, absorb, and apply new technologies. Organizational learning capability thus becomes an important boundary condition influencing the effectiveness of AI empowerment [36]. Organizational learning capability refers to a firm’s ability to acquire, absorb, integrate, and apply new knowledge and technologies, reflecting its cognitive agility and knowledge transformation capacity in response to environmental changes. Organizations with strong learning capability can quickly comprehend the logic and functions of AI systems, effectively integrate AI-generated information into supervisory processes and governance practices, and thereby amplify the effect of AI empowerment on information transparency [37]. In contrast, organizations with weak learning capability may experience “technological stagnation,” where AI-generated information is underutilized due to limited absorptive and re-creative mechanisms, weakening its potential impact on transparency [38]. Based on this reasoning, organizational learning capability strengthens the absorption and transformation processes of AI empowerment, moderating the relationship between AI empowerment and information transparency.

When organizational learning capability is high, AI empowerment more effectively promotes information flows and is positively associated with information-sharing levels, resulting in smoother organizational information-processing mechanisms [39]. Conversely, when learning capability is low, the effect of AI empowerment is constrained, and the improvement in transparency is weakened [40]. Therefore, it is posited that organizational learning capability positively moderates the relationship between AI empowerment and information transparency. Based on this reasoning, the following hypothesis is proposed [41]:

H4: Organizational learning capability moderates the relationship between AI empowerment and information transparency.

AI empowerment is positively associated with information-processing efficiency through technological means and indirectly associated with supervisory performance by increasing information transparency. However, the effect of AI empowerment varies across organizations, depending on their learning capability. As noted earlier, organizational learning capability refers to the systematic capacity to acquire, absorb, integrate, and apply new knowledge and technologies, reflecting cognitive adaptability and knowledge transformation efficiency when facing informational complexity [36]. Organizations with high learning capability can quickly grasp the operational logic of AI systems, internalize AI-derived analytical outcomes into supervisory decision-making, and thereby promote information sharing, communication, and disclosure, significantly improving transparency [39]. In contrast, organizations with low learning capability may exhibit “technological inertia,” failing to fully absorb AI-generated information, which constrains information flow efficiency and diminishes the improvement in transparency [37].

This moderating mechanism further influences the indirect pathway through which AI empowerment affects supervisory performance via information transparency. When organizational learning capability is strong, AI empowerment not only directly associated with information processing capability but also, through higher transparency, improves the quality of information available to supervisory boards, leading to more timely and accurate supervisory judgments and ultimately improving supervisory performance [37]. Conversely, when learning capability is weak, even with AI empowerment, limitations in information generation and dissemination restrict supervisory boards’ access to accurate and timely data, weakening the mediating effect of transparency on the AI–performance relationship [40]. Thus, organizational learning capability not only moderates the direct effect of AI empowerment on information transparency but also influences the strength of its indirect effect on supervisory performance through transparency. This implies that the pathway through which AI empowerment is positively associated with supervisory performance differs across organizations depending on their learning capability. Based on this reasoning, the following hypothesis is proposed:

H5: Organizational learning capability moderates the mediating effect of information transparency.

## 3. Method

### 3.1 Procedure

This study employed a questionnaire survey to collect data based on the subjective perceptions of supervisory board members and corporate managers, examining the relationships among AI empowerment, information transparency, organizational learning capability, and supervisory board effectiveness. The research process lasted for approximately two months and comprised four stages: questionnaire design, pilot testing, formal survey, and data organization, with the aim of ensuring data representativeness and reliability.

During the preparatory stage, the research team developed a preliminary questionnaire according to the research model and variable definitions. The questionnaire design followed the principles of simplicity and relevance, and all items were measured using a five-point Likert scale (1 representing “strongly disagree” and 5 representing “strongly agree”). To ensure linguistic clarity and content validity, the research team conducted multiple rounds of internal discussion and expert review before the formal survey. Items with unclear or redundant wording were revised and simplified. After several modifications, the finalized questionnaire consisted of four core sections—AI empowerment, information transparency, organizational learning capability, and supervisory board effectiveness—along with several control variables.

The formal data collection was conducted between June and September 2025. To ensure sample diversity and representativeness, both online and offline methods were used. The online questionnaire was distributed through the Wenjuanxing platform to corporate contacts, who forwarded it to supervisory board members and relevant managers. Offline surveys were administered by research assistants at corporate meetings or training sessions, where respondents were guided through the completion process. The sample covered multiple industries, including manufacturing, information technology, finance, energy, and transportation, and included state-owned enterprises, private firms, and foreign-invested companies, resulting in a comprehensive industry structure. Regarding the geographical and organizational coverage of the sample, the survey was designed to capture responses from firms located in different regions of China rather than from a single local area. The online questionnaires distributed via the Wenjuanxing platform were accessible nationwide and were completed by respondents from multiple provinces and regions. The offline surveys conducted by research assistants were implemented across different firms and occasions (e.g., corporate meetings and training sessions) and were not limited to a single organization or location. To reduce the risk that a large number of questionnaires might originate from only a few firms, particular attention was paid during data screening to ensure diversity in organizational sources.

To reduce potential methodological bias associated with single-wave data collection and to enhance the rigor of causal inference among the study variables, this research adopted a time-lagged survey design. Specifically, data were collected in three stages. In the first stage (July 1, 2025), the research team collected information on AI empowerment as well as respondents’ demographic characteristics. In the second stage (July 16, 2025), information transparency was measured. In the third stage (August 1, 2025), supervisory board effectiveness was assessed. A total of 520 questionnaires were collected in the first stage, 430 questionnaires in the second stage, and 390 questionnaires in the third stage. At each stage, questionnaires were administered using a combination of online and offline methods, and unique identifiers were assigned to ensure accurate matching of responses across different time points. During data cleaning, only those respondents who completed questionnaires at all three stages were retained. As a result, among the 390 matched questionnaires collected in the third stage, 382 valid responses were retained and constituted the final sample used for subsequent empirical analyses. This phased data collection design helps to alleviate concerns related to common method bias and potential reverse causality, thereby enhancing the robustness of the study’s findings. The final sample of 382 valid responses was drawn from multiple companies rather than a small number of large enterprises, thereby limiting the potential bias caused by firm-specific organizational cultures or unusually strong AI-oriented cultures. The respondents were nested within 128 organizations, with an average of 2.98 respondents per organization. This multi-respondent design enabled the assessment of within-organization agreement and provided a basis for aggregating individual responses to the organizational level. Overall, the sample reflects a cross-organizational and cross-regional composition, which is positively associated with the generalizability of the findings. Participation in this study was entirely voluntary. Prior to data collection, all participants were informed about the purpose of the study, the anonymity of their responses, and their right to withdraw at any time without any negative consequences. Informed consent was obtained from all participants before completing the questionnaire. For online surveys, consent was obtained electronically through the survey platform before participants proceeded to the questionnaire. For offline surveys, written consent was obtained prior to participation. No financial incentives or compensation were provided. All data were collected and processed anonymously to ensure the confidentiality and privacy of participants.

The time-lagged design adopted in this study was intended to reduce common method bias and strengthen temporal ordering among the focal variables. Following prior research on organizational cognition, technology adoption, and governance processes, a two-week interval was considered appropriate because the focal constructs examined in this study primarily reflect managerial perceptions of organizational practices rather than objective changes in organizational structures. Specifically, AI empowerment influences the accessibility, integration, and utilization of governance-related information on a continuous basis. Improvements in information transparency can therefore become perceptible to supervisory board members and senior managers within a relatively short period after exposure to AI-enabled governance processes. Similarly, supervisory board effectiveness in this study captures perceived monitoring quality, risk identification capability, and governance responsiveness rather than long-term organizational outcomes. Consequently, changes in information processing and supervisory practices are expected to emerge more rapidly than structural or financial performance outcomes. Consistent with prior time-lagged studies in organizational behavior and information systems research, the two-week interval was designed to establish temporal precedence while minimizing sample attrition and memory decay. Therefore, the selected temporal framework is theoretically appropriate for examining the proposed relationships among AI empowerment, information transparency, and supervisory board effectiveness.

Consistent with prior research in corporate governance and organizational studies, this study adopts a key-informant approach. Supervisory board members and senior managers are well positioned to provide reliable evaluations of organizational-level constructs such as AI empowerment, information transparency, and supervisory board effectiveness, as these constructs reflect institutionalized governance practices and shared supervisory processes. The focus of the survey is therefore on respondents’ assessments of organizational realities rather than on their individual experiences. Because the theoretical model of this study is specified at the organizational level whereas data were collected from individual supervisory board members and senior managers, it was necessary to establish whether respondents within the same organization shared sufficiently similar perceptions to justify data aggregation. Within-group agreement (rwg) and intraclass correlation coefficients (ICC(1) and ICC(2)) were calculated for all focal constructs. The average within-group agreement values exceeded the recommended threshold of 0.70, indicating substantial consensus among respondents from the same organization. Specifically, the rwg values were 0.75 for AI empowerment, 0.83 for information transparency, 0.91 for organizational learning capability, and 0.84 for supervisory board effectiveness. In addition, the ICC(1) values ranged from 0.17 to 0.25, indicating meaningful between-organization variance. The ICC(2) values ranged from 0.38 to 0.50, indicating relatively modest reliability of the organizational means, which may partly reflect the small number of respondents per organization. These results provide empirical support for aggregating individual responses to represent shared organizational perceptions. Accordingly, all substantive analyses were conducted at the organizational level.

To further ensure data quality and authenticity, the questionnaire introduction explicitly informed respondents that the research was conducted solely for academic purposes, that all responses would remain strictly anonymous, and that no enterprise or personal information would be disclosed. This approach helped to reduce social desirability bias and defensive responding. After data collection, the research team used SPSS 26.0 for initial data screening and cleaning, including handling missing values, detecting outliers, and checking logical consistency. Normality tests and extreme-value screening were conducted, resulting in a final dataset of 382 valid samples for subsequent analysis. The final matched sample comprised 382 respondents nested within 128 firms. Respondent-level data were used to calculate scale scores and assess the appropriateness of aggregation. We then averaged respondents’ scale scores within each firm to construct a firm-level dataset containing 128 observations. The firm, rather than the individual respondent, was the unit of analysis for the hypothesis tests.

In summary, the data collection process strictly followed scientific research protocols. Through pilot testing, phased implementation, random sampling, and multiple validation steps, the study ensured both the breadth of the sample and the reliability of the data, providing a robust empirical foundation for subsequent measurement and model analysis.

This study was conducted in accordance with the Declaration of Helsinki. Ethical review and approval were waived for this study according to Article 32 of the Measures for Ethical Review of Life Science and Medical Research Involving Human Beings of the People’s Republic of China (effective February 18, 2023; available at: https://www.nhc.gov.cn/qjjys/c100016/202302/6b6e447b3edc4338856c9a652a85f44b.shtml). Informed consent was obtained from all subjects involved in the study.

### 3.2 Measures

All variables in this study were measured through a questionnaire approach. Scale items were adapted from established measures in prior domestic and international research and were localized to fit the context of supervisory boards in Chinese enterprises. Each variable was rated on a five-point Likert scale (1 = “strongly disagree,” 5 = “strongly agree”), with higher scores indicating stronger agreement or perception by respondents.

AI Empowerment: AI empowerment measures the degree to which the adoption of AI technologies is associated with firms’ capabilities in governance and supervision. This study adapted the three-item scale developed by Medcof [42]. Although the original scale was developed before the emergence of contemporary AI technologies, it was selected because it captures the underlying concept of technology-enabled decision support and cognitive enhancement in organizational processes. Following recent AI governance research, the wording of the original items was revised to explicitly reflect AI-enabled supervisory applications, including algorithm-based analysis, intelligent decision support, and AI-assisted governance practices. Thus, the adapted scale is intended to capture AI empowerment rather than general technology usage or routine information system support. To assess the appropriateness of the adapted measure, reliability and validity analyses were conducted. The results indicated satisfactory psychometric properties. The scale demonstrated high internal consistency (Cronbach’s α = 0.96). Furthermore, the confirmatory factor analysis showed that all item loadings exceeded the recommended threshold and loaded significantly on the intended construct, supporting convergent validity. These results suggest that the adapted measure provides a reliable and valid assessment of AI empowerment in the context of contemporary corporate governance and supervisory activities.

Information Transparency: Information transparency captures the openness and accuracy of information generation, transmission, and sharing within firms. This study adapted the scales developed by Bushman et al. [43] and Cao et al. [44], modifying them to fit the Chinese corporate supervision context. A representative item is “Operational and financial information within the company is transmitted to the supervisory board in a timely and accurate manner.” The Cronbach’s α coefficient for this variable was 0.98, demonstrating satisfactory internal consistency.

Organizational Learning Capability: Organizational learning capability assesses a firm’s ability to acquire, integrate, and apply new knowledge. This study employed the 12-item scale developed by Jerez-Gómez et al. [37]. A representative item is “Management actively supports learning and knowledge-sharing activities.” The Cronbach’s α coefficient for this variable was 0.99, indicating high internal consistency and reliability.

Supervisory Board Effectiveness: Supervisory board effectiveness measures the actual performance of the supervisory board in fulfilling its oversight responsibilities, preventing risks, and improving governance outcomes. This study drew on the scale developed by Minichilli et al. [45] and localized it to the Chinese context with appropriate simplification. A representative item is “The supervisory board can promptly detect and correct potential violations or risky behaviors of the management.” The Cronbach’s α coefficient for this variable was 0.98, reflecting high reliability and internal consistency. Consistent with prior governance research, supervisory board effectiveness was operationalized as respondents’ evaluations of the board’s monitoring quality, risk identification capability, and governance performance. Although perceptual measures are widely used when objective governance outcomes are difficult to obtain, the present study acknowledges that such assessments may not fully capture all dimensions of actual supervisory effectiveness.

Control Variables: Because the hypothesis-testing models were estimated at the firm level, individual respondent demographics were not entered directly into the regressions. Instead, we summarized the characteristics of the participating respondents within each firm. Gender was represented by the proportion of male respondents; age, organizational tenure, and education were represented by the firm-level means of their respective coded categories. These variables describe the composition of the participating informants, rather than the demographic composition of each firm’s entire workforce.

## 4. Results

### 4.1 Aggregation analysis

Before hypothesis testing, aggregation analyses were conducted to verify whether individual responses could appropriately represent organizational-level constructs. Following established procedures, within-group agreement (rwg), ICC(1), and ICC(2) were calculated for all focal variables. As shown in Table 1, all rwg values exceeded the recommended threshold of 0.70, indicating satisfactory consensus among respondents within the same organization. Furthermore, the ICC(1) values ranged from 0.17 to 0.25, suggesting that a meaningful proportion of the variance was attributable to organizational membership. The ICC(2) values ranged from 0.38 to 0.50, indicating relatively modest reliability of the aggregated organizational-level scores.

**Table 1:** Aggregation statistics.

Variable	rwg	ICC(1)	ICC(2)
AI Empowerment	0.75	0.24	0.49
Information Transparency	0.83	0.21	0.44
Organizational Learning Capability	0.91	0.25	0.50
Supervisory Board Effectiveness	0.84	0.17	0.38

Taken together, these findings provide sufficient statistical justification for aggregating individual-level responses to the organizational level and support the organizational-level interpretation adopted throughout this study.

### 4.2 Common method bias

Because the data in this study were collected through self-reported questionnaires, there is a potential risk of Common Method Bias (CMB). To ensure the reliability and internal validity of the research results, tests for common method bias were conducted prior to data analysis. Using AMOS 28.0, a confirmatory factor analysis (CFA) model was constructed by adding a common latent factor that loaded on the residuals of all measurement items. The model with the common latent factor was then compared with the original measurement model. The results showed only minimal improvements in model fit after adding the latent factor (Δχ² = 58.78, Δdf = 25, ΔCFI = 0.002, ΔTLI = 0.002, ΔRMSEA = 0.005). All changes were below the conventional threshold (ΔCFI < 0.01), suggesting that common method bias had an insignificant impact on model fit.

### 4.3 Correlation and descriptive statistics

Descriptive statistics and Pearson correlation analyses were conducted for all main variables. Table 2 presents the means, standard deviations, and correlation coefficients. The results indicate that the directions of the correlations among the main variables are consistent with the research hypotheses.

**Table 2:** Means, standard deviations, and correlation coefficients of key variables.

Variables	Mean	SD	1	2	3	4	5	6	7	8
1. Gender	0.56	0.29	1							
2. Age	3.13	0.60	−0.20*	1						
3. Tenure	2.59	0.69	0.02	0.01	1					
4. Education	2.36	0.45	0.10	0.15	0.01	1				
5. AI empowerment	3.78	0.72	0.03	−0.02	−0.05	−0.05	1			
6. Information transparency	3.84	0.69	−0.00	0.01	−0.11	0.13	0.45^***^	1		
7. Organizational learning capability	3.91	0.75	0.07	−0.03	−0.02	0.03	0.43^***^	0.39^***^	1	
8. Supervisory board effectiveness	3.88	0.71	0.16	−0.07	−0.07	0.08	0.43^***^	0.48^***^	0.43^***^	1

Notes: N = 128 firms. Gender represents the proportion of male respondents within each firm; age, tenure, and education represent firm-level means of their respective coded categories. * *p* < .05, ** *p* < .01, *** *p* < .001.

AI empowerment is significantly and positively correlated with information transparency (*r* = 0.45, *p* < 0.001), suggesting that the application of AI technologies facilitates internal information flow within firms. A significant positive correlation also exists between AI empowerment and supervisory board effectiveness (*r* = 0.43, *p* < 0.001), indicating that the use of AI tools is positively associated with the efficiency of supervisory activities. The correlation between information transparency and supervisory board effectiveness is 0.48 (*p* < 0.001), supporting the inference that information transparency may play a mediating role between the two.

Moreover, organizational learning capability is positively correlated with both AI empowerment (*r* = 0.43, *p* < 0.001) and information transparency (*r* = 0.39, *p* < 0.001), implying that learning-oriented organizations are better able to leverage the supervisory value of AI empowerment. The correlations between the control variables (gender, age, tenure, and education level) and the main variables are relatively low and do not significantly influence the overall model.

In sum, the correlations among variables are moderate and no multicollinearity issues are detected, providing a sound basis for subsequent reliability, validity, and structural equation modeling analyses.

### 4.4 Confirmatory factor analysis

A Confirmatory Factor Analysis (CFA) was conducted on the four main latent variables—AI Empowerment, Information Transparency, Organizational Learning Capability, and Supervisory Board Effectiveness—to assess the convergent and discriminant validity of the measurement model. The results are presented in Table 3. The four-factor model demonstrated a satisfactory overall model fit (χ² = 368.36, *df* = 269, χ²/*df* = 1.37, CFI = 0.995, TLI = 0.994, IFI = 0.995, RMSEA = 0.031, GFI = 0.930, SRMR = 0.014). All fit indices met the commonly accepted thresholds (χ²/*df* < 3, CFI, TLI, and IFI > 0.90, RMSEA < 0.08), indicating that the measurement model fits the data well. In contrast, the single-factor model, in which all four latent constructs were combined into one factor, exhibited a significantly poorer fit (χ²/*df* = 29.50, CFI = 0.567, TLI = 0.528, RMSEA = 0.273, GFI = 0.361, SRMR = 0.251). These results further confirm the discriminant validity of the four-factor measurement model adopted in this study. Detailed measurement model results are provided in S1 Appendix.

**Table 3:** Results of Confirmatory Factor Analysis (CFA).

Model	χ²	*df*	χ²/*df*	CFI	TLI	IFI	RMSEA	GFI	SRMR
Four-factor model (AI Empowerment, Information Transparency, Organizational Learning Capability, Supervisory Board Effectiveness)	368.36	269	1.37	0.995	0.994	0.995	0.031	0.930	0.014
Three-factor model (AI Empowerment and Information Transparency combined)	1704.25	272	6.27	0.921	0.913	0.921	0.118	0.786	0.100
Two-factor model (AI Empowerment, Information Transparency, and Organizational Learning Capability combined)	4841.19	274	17.67	0.748	0.724	0.748	0.209	0.501	0.196
One-factor model (all items loaded on a single factor)	8111.24	275	29.50	0.567	0.528	0.568	0.273	0.361	0.251

### 4.5 Hypothesis testing

The regression, mediation, moderation, and conditional indirect-effect analyses were conducted using the 128 firm-level observations. Each firm contributed one observation to these models; the 382 respondent records were not treated as independent observations in hypothesis testing. Continuous predictors were mean-centered before the interaction term was calculated. We did not estimate multilevel random-intercept models.

Hypothesis 1 proposes that AI empowerment has a positive effect on information transparency. The regression results are presented in Table 4. As shown in the table, AI empowerment is positively and significantly associated with information transparency (*β* = 0.46, *p* < 0.001). This result indicates that higher levels of AI empowerment enhance the transparency and availability of information within organizations, thereby supporting Hypothesis 1.

**Table 4:** Regression results for H1.

Variable	Information Transparency
Gender	−0.03 (0.08)
Age	−0.01 (0.08)
Tenure	−0.09 (0.08)
Education	0.16 (0.08)
AI Empowerment	0.46*** (0.08)
R²	0.24
Adjusted R²	0.21
F	7.55***

Notes: N = 128 firms. Standardized coefficients are reported; standard errors are in parentheses. * *p* < 0.05, ** *p* < 0.01, *** *p* < 0.001.

Hypothesis 2 predicts that information transparency positively influences supervisory board effectiveness. The regression results reported in Table 5 show that information transparency has a significant positive effect on supervisory board effectiveness (*β* = 0.47, *p* < 0.001). This finding suggests that increased transparency enables supervisory boards to perform their monitoring and oversight functions more effectively. Therefore, Hypothesis 2 is supported.

**Table 5:** Regression results for H2.

Variable	Supervisory Board Effectiveness
Gender	0.15 (0.08)
Age	−0.05 (0.08)
Tenure	−0.02 (0.08)
Education	0.01 (0.08)
Information Transparency	0.47*** (0.08)
R²	0.25
Adjusted R²	0.22
F	8.30***

Notes: N = 128 firms. Standardized coefficients are reported; standard errors are in parentheses. * *p* < 0.05, ** *p* < 0.01, *** *p* < 0.001.

Hypothesis 3 examines whether information transparency mediates the relationship between AI empowerment and supervisory board effectiveness. The mediation results are shown in Table 6. When information transparency is included in the model, the coefficient of AI empowerment on supervisory board effectiveness decreases but remains statistically significant, while information transparency exhibits a strong positive effect on supervisory board effectiveness. Further bootstrap analysis confirms that the indirect effect of AI empowerment on supervisory board effectiveness through information transparency is significant, indicating a partial mediation effect. These results provide support for Hypothesis 3.

**Table 6:** Mediation analysis for H3.

Variable	Supervisory Board Effectiveness
Gender	0.13 (0.08)
Age	−0.05 (0.08)
Tenure	−0.02 (0.08)
Education	0.04 (0.08)
AI Empowerment	0.27** (0.09)
Information Transparency	0.35*** (0.09)
R²	0.31
Adjusted R²	0.28
F	9.10***

Notes: N = 128 firms. Standardized coefficients are reported; standard errors are in parentheses. * *p* < .05, ** *p* < .01, *** *p* < .001.

Hypothesis 4 proposes that organizational learning capability moderates the relationship between AI empowerment and information transparency. The moderation analysis results are reported in Table 7. The interaction term between AI empowerment and organizational learning capability is positive and statistically significant (*β* = 0.12, *p* < 0.001), indicating that the positive effect of AI empowerment on information transparency is stronger in organizations with higher learning capability. Thus, Hypothesis 4 is supported.

**Table 7:** Moderation analysis for H4.

Variable	Information Transparency
Gender	−0.04 (0.08)
Age	−0.01 (0.08)
Tenure	−0.09 (0.08)
Education	0.15 (0.08)
AI Empowerment	0.36*** (0.09)
Organizational Learning Capability	0.25** (0.09)
AI Empowerment × OLC	0.12*** (0.02)
R²	0.29
ΔR²	0.23
F	6.84***

Notes: N = 128 firms. Standardized coefficients; standard errors in parentheses. * *p* < .05, ** *p* < .01, *** *p* < .001.

Hypothesis 5 predicts that organizational learning capability moderates the indirect effect of AI empowerment on supervisory board effectiveness through information transparency. The results of the conditional indirect effect analysis are reported in Table 8. As shown in the table, when organizational learning capability is high (+1 SD), the indirect effect of AI empowerment on supervisory board effectiveness through information transparency is positive and statistically significant (indirect effect = 0.14, 95% CI = [0.05, 0.26]). Similarly, when organizational learning capability is low (−1 SD), the indirect effect is also statistically significant (indirect effect = 0.10, 95% CI = [0.02, 0.21]). However, the difference in the indirect effects across levels of organizational learning capability is not statistically significant, as indicated by the index of moderated mediation (index = 0.024, 95% CI = [−0.038, 0.092]), whose confidence interval includes zero. These results suggest that organizational learning capability does not significantly moderate the indirect effect of AI empowerment on supervisory board effectiveness through information transparency. Therefore, Hypothesis 5 is not supported.

**Table 8:** Conditional indirect effects for H5.

Organizational Learning Capability	Indirect Effect	95% CI
Low (−1 SD)	0.10	[0.02, 0.21]
High (+1 SD)	0.14	[0.05, 0.26]

## 5. Discussion

In the context of deepening digitalization and intelligent governance, Artificial Intelligence (AI) has become a key driver of innovation in corporate governance systems. Drawing on Information Processing Theory (IPT), this study constructed a mediated model of “AI Empowerment–Information Transparency–Supervisory Board Effectiveness” and introduced Organizational Learning Capability as a moderating variable, thereby developing a moderated mediation model. Through theoretical reasoning and empirical analysis, several major findings have emerged.

First, AI empowerment has a significant positive impact on supervisory board effectiveness. The introduction of AI technologies is positively associated with organizational efficiency in information collection, analysis, and integration, while strengthening supervisory boards’ ability to identify and assess risks in complex governance environments [46]. Through algorithmic learning, intelligent early warning, and data visualization, AI empowerment effectively reduces information asymmetry, allowing supervisory boards to access more timely and accurate operational information, thereby improving the scientific rigor and effectiveness of supervision [47]. This finding validates the core premise of Information Processing Theory that organizational performance depends on the fit between information-processing capacity and task complexity.

Second, information transparency plays a significant mediating role between AI empowerment and supervisory board effectiveness. AI empowerment does not directly enhance supervisory performance but instead improves governance outcomes by optimizing information flow and sharing mechanisms within the organization [48]. Specifically, AI applications facilitate timely disclosure, data sharing, and transparent communication, enabling supervisory bodies to make decisions based on sufficient and reliable information [49]. This finding uncovers the information-based mechanism through which AI empowerment contributes to governance performance, enriching theoretical understanding in the field of digital governance.

Third, organizational learning capability positively moderates the relationship between AI empowerment and information transparency. Organizations with strong learning capability can more effectively absorb and transform the knowledge and insights generated by AI systems, embedding them into supervisory decision-making processes and thereby amplifying the effect of AI empowerment on information transparency [50]. In contrast, organizations with weak learning capability, despite possessing advanced technologies, fail to fully leverage AI’s potential due to the lack of absorptive and re-creative mechanisms [51]. As a result, improvements in information transparency remain limited. This finding suggests that organizational learning capability is not only a prerequisite for effective AI adoption but also a crucial bridge linking technological empowerment to governance performance.

Finally, although organizational learning capability was theoretically expected to strengthen the indirect effect of AI empowerment on supervisory performance through information transparency, the moderated mediation effect was not statistically significant. The indirect effect remained positive and significant at both high and low levels of organizational learning capability, but the difference between these conditional indirect effects was not significant. This suggests that the mediating role of information transparency is relatively stable across different levels of organizational learning capability, rather than being substantially contingent on organizational learning capability. Although prior research suggests that stronger learning capability may facilitate the absorption and application of AI-generated information [52], while weaker learning capability may constrain organizations’ ability to transform technological inputs into effective governance practices [53], the present findings do not provide sufficient evidence that these differences significantly alter the indirect pathway from AI empowerment to supervisory performance through information transparency.

Although the theoretical model is grounded in Information Processing Theory and the time-lagged design provides some temporal separation among variables, the present study cannot establish definitive causal relationships. The observed associations should therefore be interpreted with appropriate caution. Reverse causality and omitted variable bias remain plausible alternative explanations despite the additional robustness analyses conducted in this study. Future research employing longitudinal panel data, experimental designs, or natural experiments would be valuable for strengthening causal inference and validating the proposed relationships. Furthermore, because supervisory board effectiveness was measured using perceptual assessments rather than objective governance indicators, the observed relationships should be interpreted as associations among organizational perceptions rather than definitive causal effects.

In sum, this study develops and tests a moderated mediation model based on Information Processing Theory, revealing the mediating role of information transparency and the moderating role of organizational learning capability in the relationship between AI empowerment and supervisory board effectiveness. Although the proposed moderated mediation effect was not supported, the findings provide a more nuanced understanding of the mechanisms and boundary conditions associated with AI-enabled supervision.

### 5.1 Theoretical contributions

First, this study advances the literature on AI and corporate governance by moving beyond established technology-performance frameworks and explaining AI empowerment as an organizational information-processing capability. Prior research has often examined digital technologies or AI adoption in terms of direct performance consequences, treating technology primarily as an external resource or operational tool [54,55]. In contrast, this study draws on Information Processing Theory to conceptualize AI empowerment as a mechanism that expands the organization’s capacity to collect, interpret, integrate, and use governance-related information. This perspective shifts the analytical focus from whether AI improves performance to how AI reshapes the informational foundations of supervisory governance [56]. By situating AI empowerment within supervisory board activities, this study extends Information Processing Theory from traditional organizational design and decision-making contexts to AI-enabled corporate supervision. In doing so, it highlights the role of AI not simply as a technological input but as a governance-enabling information-processing infrastructure, thereby enriching theoretical understanding of intelligent governance and digital supervision [57,58].

Second, this study contributes to governance research by identifying information transparency as a key explanatory mechanism linking AI empowerment to supervisory board effectiveness. Existing technology-performance frameworks often assume that technological adoption produces performance gains through efficiency improvement, while paying less attention to the internal governance processes through which such gains are realized. This study shows that the value of AI empowerment in corporate supervision lies not only in the use of advanced analytical tools but also in its association with more transparent, timely, and traceable information flows. By introducing information transparency as a mediating mechanism, the study explains how AI-enabled information processing can reduce information asymmetry, improve the availability of governance-relevant information, and support supervisory boards in making more informed judgments [59,60]. This finding advances existing AI governance research by opening the “black box” between AI adoption and governance outcomes and by demonstrating that information transparency is a critical channel through which AI empowerment is associated with supervisory effectiveness.

Third, this study extends research on organizational learning by clarifying its boundary-setting role in AI-enabled governance. Established technology-performance perspectives often imply that the benefits of technology depend mainly on technological sophistication or adoption intensity. However, the present study shows that the governance value of AI empowerment is also associated with whether organizations possess sufficient learning capability to absorb, interpret, and transform AI-generated information. By incorporating organizational learning capability as a moderator, this study provides a more nuanced explanation of why similar levels of AI empowerment may be associated with different levels of information transparency across organizations [53,61]. Organizations with stronger learning capability are better able to integrate AI-derived insights into supervisory routines, thereby strengthening the relationship between AI empowerment and information transparency. Conversely, organizations with weaker learning capability may be less effective in translating AI-enabled information into transparent governance processes.

Importantly, however, the present findings do not support the proposed moderated mediation effect. Although organizational learning capability significantly moderates the relationship between AI empowerment and information transparency, it does not significantly alter the indirect effect of AI empowerment on supervisory board effectiveness through information transparency. This distinction refines the proposed theoretical framework by suggesting that organizational learning capability shapes the initial translation of AI empowerment into improved information transparency, but does not necessarily determine the strength of the entire indirect pathway to supervisory effectiveness. In this sense, the findings contribute to a more differentiated understanding of AI-enabled governance by showing that the influence of organizational learning capability may be concentrated at a specific stage of the information-processing process rather than extending uniformly across the full mediation mechanism. This nuanced perspective integrates technological capability, information mechanisms, and organizational learning while also identifying the limits of the proposed boundary condition [52,61].

### 5.2 Practical implications

First, organizations should strengthen AI empowerment and build intelligent supervisory support systems. The findings show that AI technologies significantly associated with information-processing capability and supervisory performance. Firms should strategically integrate AI into governance systems and treat it as a core tool for supervisory decision-making rather than as a purely technical investment. Specifically, AI-driven data monitoring, intelligent warning, and automated analytics systems can be introduced into supervisory processes to track financial, risk, and compliance information in real time, enabling supervisory boards to identify anomalies quickly and shorten feedback cycles, thereby shifting from reactive supervision to proactive early warning. Moreover, firms should promote collaboration between supervisory boards and information technology departments, establishing two-way communication mechanisms between technological expertise and governance needs, so that AI empowerment genuinely supports supervisory decision-making and risk identification.

Second, organizations should improve information disclosure and communication mechanisms to enhance overall transparency. Information transparency is the key link through which AI empowerment translates into governance performance. Firms should optimize disclosure systems, promote real-time information sharing across hierarchical levels, and eliminate the traditional “information silo” phenomenon. Management should build open data platforms and multidimensional reporting systems that allow supervisory boards to make judgments based on accurate, real-time data. Furthermore, firms should strengthen oversight of disclosure quality, ensuring that information is not only accessible but also usable. Externally, companies should institutionalize a culture of transparency by encouraging open, truthful, and responsible information exchange, thereby fostering trust among investors, regulators, and the public, and creating a virtuous cycle between internal transparency and external credibility.

Third, organizations should cultivate a learning-oriented culture to enhance the absorption and transformation of AI empowerment. The study reveals that organizational learning capability is a critical condition determining whether AI empowerment is positively associated with transparency and supervisory performance. Firms should therefore prioritize the development of learning mechanisms during their transition toward intelligent governance. Supervisory board members should be encouraged to update their knowledge base and enhance their data literacy and technological sensitivity. Management can facilitate learning through training, workshops, and cross-departmental exchanges to promote the integration of AI tools with governance practices. An open and inclusive learning environment should also be fostered, encouraging experimentation and innovation so that practical experiences with AI empowerment are continually accumulated and disseminated. By cultivating a learning-oriented organizational culture, firms can internalize AI capabilities and continuously strengthen their role in information processing and supervisory decision-making, ultimately achieving sustainable competitive advantage in the digital governance era.

### 5.3 Limitations and future research

Although this study provides systematic theoretical and empirical insights into the relationships among AI empowerment, information transparency, supervisory board effectiveness, and organizational learning capability, several limitations remain. Another limitation concerns the geographical context of the study. The sample was drawn exclusively from Chinese firms operating within a specific institutional, regulatory, and cultural environment. Consequently, the findings should be interpreted primarily within the Chinese corporate governance context. Differences in governance structures, regulatory systems, and digital transformation practices across countries may influence the relationships examined in this study. Future research is encouraged to replicate the proposed model in different national and institutional settings to assess the generalizability of the findings.

First, regarding research design, this study adopted a three-wave time-lagged survey design, which identifies correlations among variables but cannot fully capture the dynamic effects of AI empowerment on supervisory performance over time. The implementation of AI and the optimization of information-processing mechanisms are typically gradual processes that may involve time lags and stage-specific effects. Future studies could employ longitudinal or panel data designs to examine the long-term interactions between AI empowerment and governance performance, thereby enhancing causal inference.

Second, regarding measurement, the constructs in this study were primarily assessed using perceptual measures reported by supervisory board members and senior managers. Although the measurement scales were adapted from well-established instruments and demonstrated satisfactory reliability and validity, the assessment of supervisory board effectiveness relied largely on subjective evaluations rather than objective governance outcomes. Consequently, the results may be influenced by respondent leniency, self-enhancement tendencies, or social desirability bias. Several procedural and statistical remedies were adopted to mitigate these concerns. First, data were collected from two categories of organizational informants—supervisory board members and senior managers—who occupy different governance roles but possess direct knowledge of supervisory practices. Second, the study employed a three-wave time-lagged design to reduce common method bias and strengthen temporal separation among the focal constructs. Third, confirmatory factor analysis, common method bias tests, and aggregation analyses all provided evidence supporting the validity of the measurement model. Nevertheless, subjective perceptions cannot fully substitute for objective indicators of governance effectiveness. Future research is encouraged to complement survey-based measures with archival and secondary governance data, such as supervisory board meeting frequency, audit expenditures, disclosure timeliness, internal control quality, regulatory compliance records, or other externally verifiable governance indicators. Such multi-source designs would further strengthen construct validity and provide a more comprehensive assessment of AI-enabled supervisory effectiveness.

Third, concerning model construction, while this study developed a moderated mediation model based on Information Processing Theory, it did not include other potential mediating variables. AI empowerment may also influence governance performance through mechanisms such as internal control quality, risk identification, or decision-support improvement. Future research could adopt multiple theoretical perspectives, such as dynamic capability theory or sociotechnical systems theory, to build more complex multiple mediation or cross-level models, thereby revealing deeper causal mechanisms of AI empowerment in corporate governance systems. Although the AI empowerment scale was adapted to reflect contemporary AI applications, future research may further develop and validate dedicated AI empowerment scales that more explicitly capture emerging AI technologies such as generative AI, large language models, and autonomous decision-support systems.

Finally, regarding sample selection, this study focused primarily on supervisory board members and senior executives from a subset of firms. The relatively concentrated sample and limited variation in industry and ownership structure may constrain external validity. Firms across different industries exhibit substantial variation in governance structures, digital transformation levels, and organizational cultures, which may lead to differing effects and pathways of AI empowerment. Future research should extend samples across industries, regions, and even national contexts to test the model’s generalizability and is positively associated with the explanatory power and universality of the findings.

## 6. Conclusion

This study investigates how AI empowerment is positively associated with supervisory board effectiveness by drawing on Information Processing Theory and developing a moderated mediation model. Using survey data collected from supervisory board members and senior executives, this research examines the roles of information transparency as a mediating mechanism and organizational learning capability as a boundary condition. The findings demonstrate that AI empowerment is positively associated with supervisory board effectiveness both directly and indirectly through enhanced information transparency. By expanding organizational information-processing capacity, AI technologies facilitate more timely, accurate, and transparent information flows, thereby enabling supervisory boards to perform monitoring tasks more effectively. Moreover, organizational learning capability strengthens the positive effect of AI empowerment on information transparency. Moreover, organizational learning capability strengthens the positive relationship between AI empowerment and information transparency. However, the findings do not support a significant moderated mediation effect, suggesting that although learning capability shapes how AI empowerment is associated with information transparency, it does not significantly alter the indirect effect of AI empowerment on supervisory board effectiveness through information transparency.

## Supporting information

S1 Appendix Measurement model results. (DOC)
