# Supplementary material for: AI empowerment and supervisory board effectiveness: Evidence from an information processing framework
Source: PLoS One. 2026 Oct 8;21(10):e0360152. doi: 10.1371/journal.pone.0360152 (PMC13649021; doi:10.1371/journal.pone.0360152)
Supplement: Supplementary file 1 [file pone.0360152.s001.doc]

**S1 Appendix.** Measurement Model Results

| **Construct** | **Code** | **Measurement Item** | **AVE** | **CR** |
| --- | --- | --- | --- | --- |
| AI Empowerment | AI1 | When making important supervisory or governance decisions, I take into account the judgment provided by AI systems. | 0.898 | 0.964 |
|  | AI2 | AI technologies assist our organization in identifying, analyzing, and interpreting governance-related information more effectively. |  |  |
|  | AI3 | AI-supported systems enhance the quality and efficiency of supervisory and governance decision-making processes. |  |  |
| Information Transparency | IT1 | Information disclosure within the organization is accurate and reliable. | 0.912 | 0.981 |
|  | IT2 | The supervisory board can easily access information needed for monitoring and governance activities. |  |  |
|  | IT3 | Information asymmetry between management and the supervisory board is effectively reduced. |  |  |
|  | IT4 | Operational and financial information within the company is transmitted to the supervisory board in a timely and accurate manner. |  |  |
|  | IT5 | Important governance-related information is shared openly among relevant organizational units. |  |  |
| Organizational Learning Capability | OLC1 | Employees are supported in applying newly acquired knowledge to their work. | 0.929 | 0.994 |
|  | OLC2 | The organization is capable of adapting to environmental and technological changes. |  |  |
|  | OLC3 | Learning is regarded as a strategic priority within the organization. |  |  |
|  | OLC4 | Management actively supports learning and knowledge-sharing activities. |  |  |
|  | OLC5 | Employees are encouraged to continuously acquire new knowledge and skills. |  |  |
|  | OLC6 | The organization systematically evaluates and improves its learning processes. |  |  |
|  | OLC7 | New ideas and innovative solutions are welcomed within the organization. |  |  |
|  | OLC8 | Learning from past successes and failures is encouraged. |  |  |
|  | OLC9 | Knowledge is effectively shared across departments and functions. |  |  |
|  | OLC10 | Employees are encouraged to question existing practices and assumptions. |  |  |
|  | OLC11 | The organization actively seeks external knowledge and best practices. |  |  |
|  | OLC12 | New knowledge is rapidly integrated into organizational operations. |  |  |
| Supervisory Board Effectiveness | SBE1 | The supervisory board can effectively analyze relevant information when performing its monitoring responsibilities. | 0.922 | 0.983 |
|  | SBE2 | The supervisory board can identify potential risks and problems in corporate operations in a timely manner. |  |  |
|  | SBE3 | The supervisory board can provide effective recommendations for improving corporate governance. |  |  |
|  | SBE4 | The supervisory board can promptly detect and correct potential violations or risky behaviors of management. |  |  |
|  | SBE5 | The supervisory board effectively fulfills its monitoring and oversight responsibilities. |  |  |

Notes: All items were measured using a five-point Likert scale ranging from 1 = strongly disagree to 5 = strongly agree. Standardized factor loadings were obtained from the confirmatory factor analysis (CFA).
